# Normal spirometry prediction equations for the Iranian population

**DOI:** 10.1186/s12890-022-02273-8

**Published:** 2022-12-12

**Authors:** Leyla Sahebi, Besharat Rahimi, Mamak Shariat, Seyyed Hosein Mousavy, Mohsen Hosseini

**Affiliations:** 1 Maternal, Fetal and Neonatal Research Center, Family Health Research Institute, Tehran University of Medical Sciences, Tehran, Iran; 2 Advanced Thoracic Research Center, Tehran University of Medical Sciences, Tehran, Iran; 3 Municipality of Tehran, Tehran, Iran; 4 School of medicine, Tehran University of Medical Science, Tehran, Iran

**Keywords:** Spirometry, Reference standards, Respiratory function tests

## Abstract

**Background:**

This study aimed to establish normative spirometric equations in a healthy population of Iranian children and adults, and compare these equations with those developed by the Caucasian Global Lung Initiative (GLI) for the first time.

**Methods:**

Spirometric data from healthy Iranian aged 4–82 years sampled in 2019 were used to derive reference equations using the generalized additive model for location (mu), shape (lambda), and scale (sigma).

**Results:**

A total of 418 females and 204 males were included in the study. Applying the GLI standards for the Iranian population resulted from the Z scores of FEV_1_, FVC, FEV_1_/FVC, and FEF_25−75%_ was not different from zero. Based on the newly calculated LLN, eleven individuals showed significant values below the LLN for FEV_1_/FVC. In all age groups, this frequency was less than 5%, except for men over 70 years of age, which was 12.5%. There are significant differences between new data and GLI for Caucasian data.

**Conclusion:**

It is recommended that the values and equations generated from this study should be used by physicians and technicians in their routine practice for the diagnosis and assessment of pulmonary disorders.

**Supplementary Information:**

The online version contains supplementary material available at 10.1186/s12890-022-02273-8.

## Introduction

Experimental diagnosis of respiratory diseases, their intensity, and prognosis are principally dependent on spirometric results [1]. Accurate interpretation of spirometry requires standardized reference values that are predicted from its population race, as well as, age, and height [2–5].

In 2012, the Global Lung Function Initiative (GLI-2012) reported normative reference values, derived from over 160,000 data points in combined datasets from 33 countries. The GLI-2012 equations provided sex, age, height, and ethnic-specific reference equations as well as the lower limit of normal (LLN) values for spirometry [5]. Although this approach included data from various countries, it did not include many populations.

Appropriateness of the GLI-2012 equations should be confirmed prior to their use for regions that are not currently covered by the reference equations  [6]. In some studies has been confirmed suitability of GLI-2012 norms for their population, for example in the Australasian [7], Norwegian [8], German [9] and French [10] populations. But, the GLI-2012 norms seem inappropriate for use in the Swedish [11], Finnish [12], and Chinese [13] populations.

Our previous study found that Caucasian GLI equations were not suitable for the Iranian population, especially children under 10 years old [14]. The lack of predictive values specific to the Iranian population may lead to the misclassification of disease. Therefore, standardization of spirometry reference values ​​is very necessary.

Respiratory scales dedicated by spirometry to each person, do not follow a linear model, and the lung volume changes according to height and age with a skewed distribution. A practical approach that has been applied for spirometric data is Generalized Additive Models for Location, Scale, and Shape (GAMLSS). GAMLSS model is a non-parametric regression equation that best fits pulmonary and spirometric measures distribution. This model is the best existing one for the prediction of pulmonary values and the prediction equations offered by GLI-2012 have been confirmed and endorsed by many international respiratory societies. [15, 16].

Simulations show that when the confounders have a non-linear association with the outcome, compared to a parametric representation, GAMLSS modelling reduce the mean squared error for the adjusted exposure effect and avoid inflation of the type I error for testing the exposure effect [17].

This, was the first study in the Iranian population that aimed to predict the standard values ​​of spirometry for Iranian reference population.

## Methods

### Design

This cross-sectional study was performed in Iran (Tehran) in 2019. This study was approved by the National Institute for Medical Research Development (NIMAD) (code: 978,931, 2019/05/28) and the Ethics Committee (code: IR.NIMAD.REC.1398.257). Conscious and written consent was obtained from all participants.

The study population was gathered from Tehran, and those who referred to local health centers- associated with Tehran Municipality- were included in the study. Overall 44 local health centers were selected by the randomized clustering method. The age range for inclusion in the study was 3–95 years. Informed consent was obtained from all participants / their legal guardians.

Healthy non-smokers between 3 and 95 years old, without a history of current airway or lung disease were included in the study. Exclusion criteria were as follows: not eligible for spirometry test and occurrence of respiratory disorders such as sputum cough, and rhinorrhea in the last 7 days.

Demographical and anthropometric variables such as sex, age, height, and weight were documented. Spirometric indices included FEV_1_, FVC, FEV_1_/FVC, and FEF_25–75%_ (Forced expiratory flow averaged over the middle portion of FVC) were measured.

The validity, repeatability, and quality control were done according to the American Thoracic Society/European Respiratory Society (ATS/ERS) recommendations [18, 19], and described in more detail in an earlier paper (first phase of this study) [14].

In this study, 418 females and 204 males in different age groups (4–82 years old) were eligible to enter the study.

### Analysis

In the earlier study [14] we measured the lower limits of normal (LLN), Z-scores and percentiles for FEV_1_, FVC, FEV_1_/FVC, and FEF_25–75%_ for each person. We determined agreement between the observed values in our population and the GLI reference values. According to the agreement by the GLI team, a mean Z-score outside the range of ± 0.5 was considered clinically significant [5, 11, 20, 21, 22]. The relationship between Z-scores and age, height, weight, and sex was examined using multiple linear regression models in the previous article [14].

In this study, GLI-2012 equations were implemented using the Generalized Additive Models for Location, Scale, and Shape (GAMLSS4.3-1) in software R (version 3.1.2; http://www.r-project.org), this method permitted the fitness of mean (M), coefficient of variance (S), and skewness (L) (Lambda–Mu–Sigma (LMS) of spirometry indices [5, 23].

The spirometry indices were modeled in males and females by age and height as explanatory variables using the Box-Cox-Cole–Green (BCG) distribution. The fittest regression models were chosen by using Schwarz Bayesian Criterion (SBC), Akaike’s Information Criterion (AIC) and assessing optimal degrees of freedom (df) for the cubic spline curve. The goodness of fit was also checked by normal Q–Q plots. Mean (M) indicates the predicted value as follows: M = exp [a + b × ln (height_cm_) + c × ln (age_year_) + M-spline] (a, b, and c are coefficients, and M-spline is an age-specific contribution from the spline function. Values of L and S were also calculated based on regression output values of Sspline and Lspline. Finally we calculated LLN as follows: LLN (5th percentage) = exp [ln (M) + ln (1 − 1.645 × L × S)/L]. Z-scores were calculated as (observed-predicted)/SD, where SD was calculated as (predicted-LLN)/1.645 [1, 23].

Agreement between Caucasian values and GLI-2012 Iranian prediction analyzed by Bland–Altman plots.

## Result

Six hundred and twenty-two Iranian participants (418 females and 204 males) aged 4–82 years were finally included in this study. The mean (range) age was 38.34 (4–82) years for men and 44.55 (4–80) years for women. The mean (SD) height for men and women were 1.72 (0.08) m and 1.58 (0.08) m over 21 years, respectively. Thirty-nine (19.2%) men and 131 (31.4%) women had a BMI ≥ 30 kg/m^2^ (Table 1). Demographical and spirometry measurements of the reference population by gender are shown in Table 1  (Table 1).

**Table 1:** Demographical and spirometry measurements of the reference population by gender

Variables	Females (414)	Males (206)
Age; mean (SD), y	45.54 (15.67)	37.78 (19.56)
Weight; mean (SD), Kg	68.61 (16.25)	74.88 (20.99)
Height; mean (SD), cm	156.18 (11.09)	168.22 (16.27)
BMI; mean (SD)	27.84 (5.74)	25.8 (5.23)
FEV1; mean (SD), l Min–Max	2.63 (0.65) 0.79–5.01	3.65 (1.04) 0.86–6.58
FVC; mean (SD), l Min–Max	3.20 (0.78) 0.9 − 0.78	4.42 (1.24) 1.15–7.5
FEV_1_/FVC; mean (SD) Min–Max	0.82 (0.70) 0.65–1.49	0.83 (0.067) 0.51-1.0
FEF_25–75%_; mean (SD), l Min–Max	3.88 (1.29) 0.49–8.13	1.51 (0.523) 0.55–2.22

*BMI* Body mass index, *FEF*_25–75%_ Forced expiratory flow at 25 and 75% of the pulmonary volume, *FEV*_1_ Forced expiratory volume in one second, *FVC* Forced expiratory vital capacity, *Min–Max* Minimum–Maximum, *SD* Standard deviation

The Caucasian GLI-2012 was applied to this sample in earlier study [14]. The mean Z-scores of FEV_1_, FVC and the FEV_1_/FVC for males and females in different age groups were higher than the Caucasian predicted values (range: 0.01 to 1.05) except for the FEV1/FVC in the age group under 21 years (range: −1.11 to − 0.09).

The Z-scores of FEV_1_, FVC, FEV_1_ /FVC, and FEF_25–75%_ distribution based on Caucasian equation by sex and age in the Iranian healthy people is accessible in Table 2.

**Table 2:** Distributions of Z-Scores based on Iranian (new) and Caucasian (GLI-2012 equations (Assumption test: Compare means with zero)

Age group(Y)	Sex M	n	FEV1		FVC		FEV1/FVC		FEF_25 − 75%_	
			Mean ± SD		Mean ± SD		Mean ± SD		Mean ± SD	
			CI 95%		CI 95%		CI 95%		CI 95%	
			Caucasian	Iranian	Caucasian	Iranian	Caucasian	Iranian	Caucasian	Iranian
< 10		13	0.524 (0.621)	0.003 (0.197)	0.721 (0.627)	0.023 (0.214)	−1.11 (1. 22)	− 0.005 (0.14)	− 0.111 (0. 824)	− 0.041 (1.013)
			0.148, 0.899	− 0.12, 0.122	0.341,1.09	− 0.107, 0.152	− 1.84, − 0.37	− 0.087, 0.077	− 0.609, 0.388	− 0.65, 0.571
	F	20	0.402 (0.904)	− 0.33 (0.539)	0.618 (0.799)	− 0.037 (0.534)	− 1.21 (1.08)	− 0.001 (0.12)	− 0.589 (0.615)	− 0.01 (0.7)
			− 0.021, 0.825	− 0.29, 0.22	0.244, 0.992	− 0.287, 0.213	− 1.72,− 0.707	− 0.061, 0.058	− 0.878,-0.301	− 0.338, 0.318
10–21	M	38	0.269 (1.29)	0.008 (0.335)	0.396 (1.28)	0.011 (0.327)	− 0.09 (1.01)	0.014 (0.13	− 0.089 (1.02)	0.044 (0.606)
			− 0.155, 0.693	− 0.102, 0.118	− 0.026, 0.818	− 0.119, 0.097	− 0.421, 0.241	− 0.06, 0.11	− 0.4250.247	− 0.155, 0.243
	F	15	0.374 (1.086)	0.042 (0.327)	0.503 (0.685)	0.034 (0.198)	− 0.323 (1.22)	0.022 (0.16)	− 0.193 (1.46)	− 0.005 (1.31)
			− 0.227, 0.975	− 0.139, 0.223	0.124 , 0.883	− 0.0756, 0.143	− 0.997, 0.35	− 0.06, 0.11	− 0.999.614	− 0.719, 0.729
22–29	M	21	0.162 (0.789)	− 0.042 (0.208)	0.292 (0.714)	− 0.03 (0.179)	0.101 (0.516)	0.009 (0.09)	− 0.437 (0. 946)	− 0.0129 (0.598)
			− 0.197, 0.521	− 0.137, 0.052	− 0.032, 0.617	− 0.112, 0.051	− 0.135, 0.335	− 0.029, 0.058	− 0.867-0.007	− 0.401, 0.144
	F	23	0.145 (0.896)	0.022 (0.214)	0.423 (1.02)	0.018 (0.232)	0.014 (1. 04)	0.007 (0.15)	− 0.664 (1.1)	0.075 (0.685)
			− 0.242, 0.532	− 0.071, 0.114	− 0.019, 0.865	− 0.083, 0.118	− 0.436, 0.465	− 0.05, 0.032	− 1.097-0.232	− 0.221, 0.371
30–39	M	43	0.461 (1.27)	− 0.005 (0.313)	0.542 (1.22)	− 0.005 (0.297)	0.231 (0.688)	0.004 (0.11)	− 0.106 (0.865)	0.029 (0.689)
			0.069, 0.854	− 0.101, 0.092	0.166, 0.917	− 0.097, 0.086	0.019, 0.443	− 0.05, 0.03	− 0.444.233	− 0.183, 0.242
	F	84	0.503 (1.24)	0.012 (0.315)	0.896 (1.29)	0.014 (0.313)	0.064 (0.665)	− 0.006 (0.09)	− 0.58 (0.865)	− 0.008 (0.617)
			0.234, 0.773	− 0.056, 0.080	0.616,1.18	− 0.053, 0.082	− 0.08, 0.209	− 0.026, 0.013	− 0.768 − 0.393	− 0.142, 0.125
40–49	M	25	0.648 (0.842)	0.033 (0.24)	0.647 (0.935)	− 0.027 (0.257)	0.420 (0.633)	0.005 (0.09)	− 0.072 (0. 769)	0.117 (0.591)
			0.30, 0.996	− 0.067, 0.132	0.261,1.03	− 0.079, 0.133	0.159, 0.681	− 0.03, 0.039	− 0.389.246	− 0.126, 0.361
	F	99	0.567 (1.16)	− 0.039 (0.038)	0.906 (1.19)	− 0.042 (0.357)	0.277 (0.774)	− 0.006 (0.11)	− 0.489 (1.08)	− 0.057 (0.881)
			0.336, 0.797	− 0.114, 0.036	0.667,1.15	− 0.113, 0.029	0.123, 0.431	− 0.028, 0.017	− 0.705 − 0.272	− 0.233, 0.118
50–59	M	29	0.594 (1.09)	0.041 (0.374)	0.618 (0.974)	0.062 (0.319)	0.417 (0.876)	0.028 (0.14)	− 0.22 (0. 888)	− 0.127 (0.81)
			0.81,1.01	− 0.102, 0.183	0.248, 0.988	− 0.06, 0.183	0.084, 0.75	− 0.034, 0 0.043	− 0.558.117	− 0.432, 0.184
	F	10	0.829 (1.11)	0.019 (0.395)	1.05 (1.48)	0.019 (0.396)	0.449 (0.784)	− 0.005 (0.11)	− 0.409 (0.905)	− 0.0524 (0.954)
		6	0.615,1.04	− 0.057, 0.096	0.76,1.33	− 0.056, 0.096	0.298, 0.599	− 0.011, 0.032	− 0.558.117	− 0.131, 0.236
60–69	M	19	0.515 (1.02)	− 0.02 (0.346)	0.301 (0.897)	− 0.097 (0.293)	0.856 (0.838)	0.054 (0.13	0.196 (1.078)	0.314 (1.344)
			0.023,1.01	− 0.187, 0.147	− 0.13, 0.733	− 0.238, 0.044	0.452,1.26	− 0.08, 0.025	− 0.323.716	− 0.333, 0.962
	F	63	0.723 (1.15)	− 0.001 (0.581)	1.02 (1.18)	0.008 (0.569)	0.32 (0.757)	0.01 (0.11)	− 0.533 (0.86)	− 0.0425 (1.17)
			0.433,1.01	− 0.147, 0.145	0.723,1.32	− 0.135, 0.151	0.132, 0.513	− 0.048, 0.010	− 0.749 − 0.316	− 0.338, 0.253
> 70	M	16	0.357 (2.23)	− 0.181 (0.928)	0.476 (2.02)	− 0.083 (0.757)	− 0.613 (1.65)	− 0.096 (0.25)	0.04 (1.28)	− 0.498 (1.73)
			− 0.829, 1.54	− 0.68, 0.314	− 0.599,1.55	− 0.49, 0.32	− 1.49, 0.269	− 0.010, 0.118	− 0.685.676	− 1.42, 0.426
	F	8	0.356 (0.822)	− 0.12 (0.495)	0.06 (0.662)	− 0.152 (0.385)	0.073 (0.983)	− 0.016 (0.16)	0.085 (1.08)	0.009 (1.93)
			− 0.331, 1.043	− 0.532, 0.295	− 0.494, 0.614	− 0.475, 0.169	− 0.748, 0.896	− 0.12, 0.149	− 0.819.989	− 1.61, 0.624

F = Female, M = male

*FEF*_25–75%_ Forced expiratory flow at 25 and 75% of the pulmonary volume, *FEV*_1_ Forced expiratory volume in one second, *FVC* Forced expiratory vital capacity, *SD* Standard deviation

### Iranian version of reference equations for spirometric values

We modeled GAMLSS regression equations for each spirometric parameter (FEV_1_, FVC, FEV_1_/FVC, and FEF_25−75%_ obtained from the study population (Look up Tables and equations are available in the supplementary material). For new (Iranian) equations, none of the Z scores (FEV_1_, FVC, FEV_1_/FVC and FEF_25−75%),_ are different from zero (by one-sample t-test analysis) in all age groups (P > 0.05). Distributions of Z-Scores based on Iranian (new) and Caucasian (GLI-2012 equations were shown in Table 2.

The obtained reference equations are used to estimate the Lower limit normal (LLN) of the spirometric parameters of FEV_1_, FVC, FEV_1_/FVC, and FEF_25−75%,_ for the Iranian population (Look up Tables are available in the supplementary material). Based on the newly calculated LLN, only five individuals showed significant values below the LLN for FEV_1_, FVC and eleven individuals for FEV_1_/FVC. This value was calculated in the Caucasian equation of 29 cases [14]. In all age groups, the frequency of Z-score for FEV_1_/FVC below the LLN was less than 5% except in men aged 70–84 years (12.5%) (Table 3). In the Caucasian equations, the Z-score of FEV_1_/FVC was significantly higher among < 21 years old (46.2% and 40.0% in males and females respectively). Frequency of FEV_1_/FVC < LLN by age and sex in Caucasian and Iranian equation is shown in Table 3.

**Table 3:** Frequency of FEV1/FVC < LLN by age and sex in Caucasian and Iranian equation

Age groups	Total (622)			Males (204)			Females (418)		
	n	Caucasian	Iranian	n	Caucasian	Iranian	n	Caucasian	Iranian
< 10	33	14 (42.4)	0	13	6 (46.2)	0	20	8 (40.0)	0
10–21	53	4 (7.5)	2 (3.78)	38	2 (5.3%)	1 (2.63)	15	2 (13.3)	1 (6.7)
22–29	44	2 (4.5)	0	21	0	0	23	2 (8.7)	0
30–39	127	1 (0.8)	1 (0.8)	43	0	0	84	1 (1.2)	`1 (1.2)
40–49	124	1 (0.8)	1 (0.8)	25	0	0	99	1 (1.0)	1 (1.0)
50–59	135	3 (2.2)	2 (1.15)	29	1 (3.4)	1 (3.4)	106	2 (1.9)	1 (0.94)
60–69	82	0	3 (3.66)	19	0	0	63	0	3 (4.8)
70–84	24	4 (16.7)	2 (8.3)	16	4 (25.0)	2 (12.5)	8	0	0

*LLN*  Lower limit of normal

Overall, residual Z-score for regression models was not beyond ± 3 for our model (the standard range for residual is ± 5). (Normal Q–Q plots (Additional file 1: Fig. S1a–h).

**Fig. 1:**
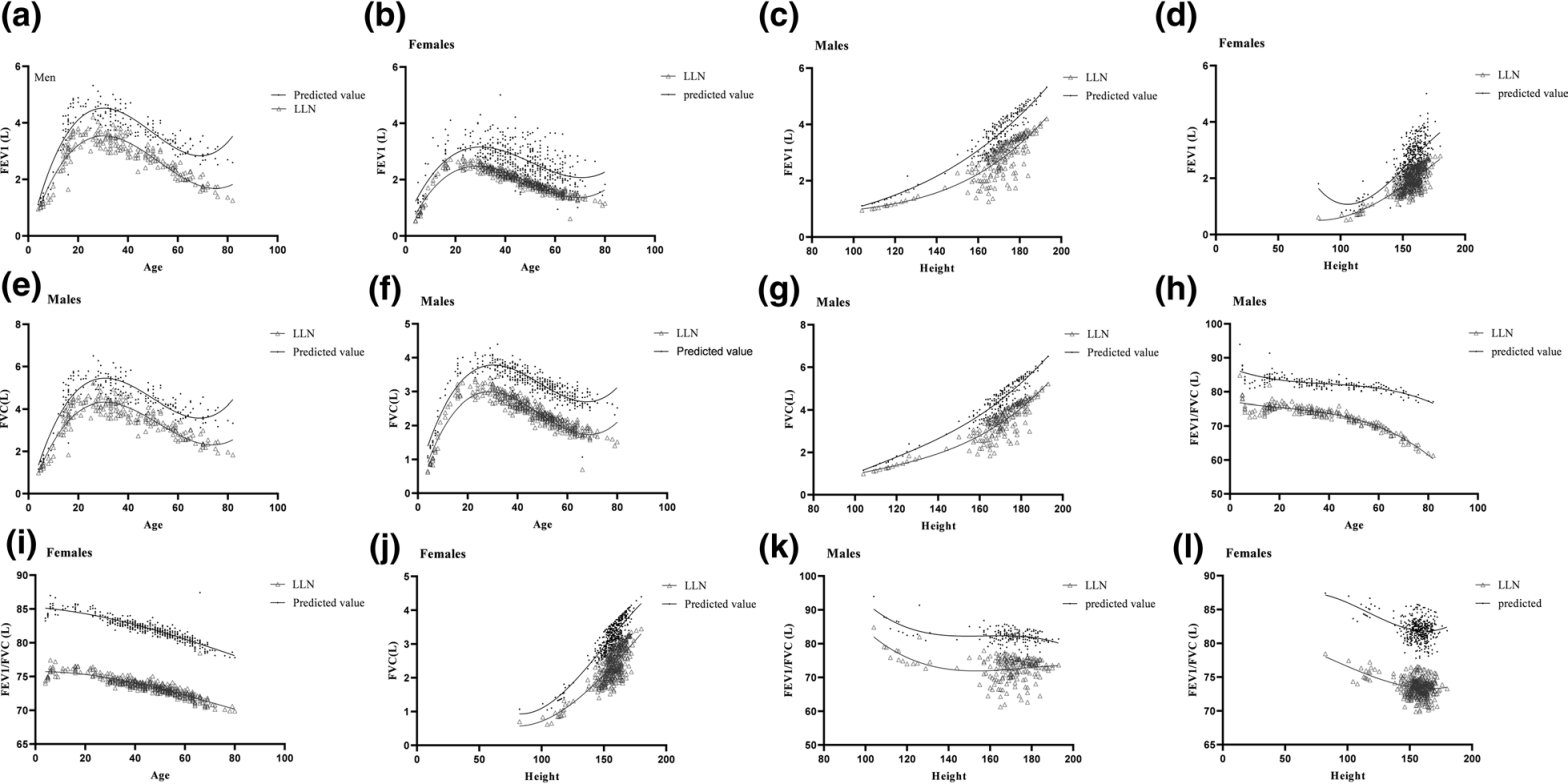
Nonlinear correlation analysis between FEV1, FVC and FEV_1_/FVC, and anthropometric parameters

We found that age and height were the main predictors of the FEV_1_ (males), FVC (males and females), and age for FEV_1_/FVC (not height) in both sex for final prediction models by nonlinear correlation analysis. The association between spirometric indices and anthropometric parameters is shown in Fig. 1a–l and Additional file 2: Table S1.

### Agreement between Caucasian values and GLI-2012 Iranian prediction

**Fig. 2:**
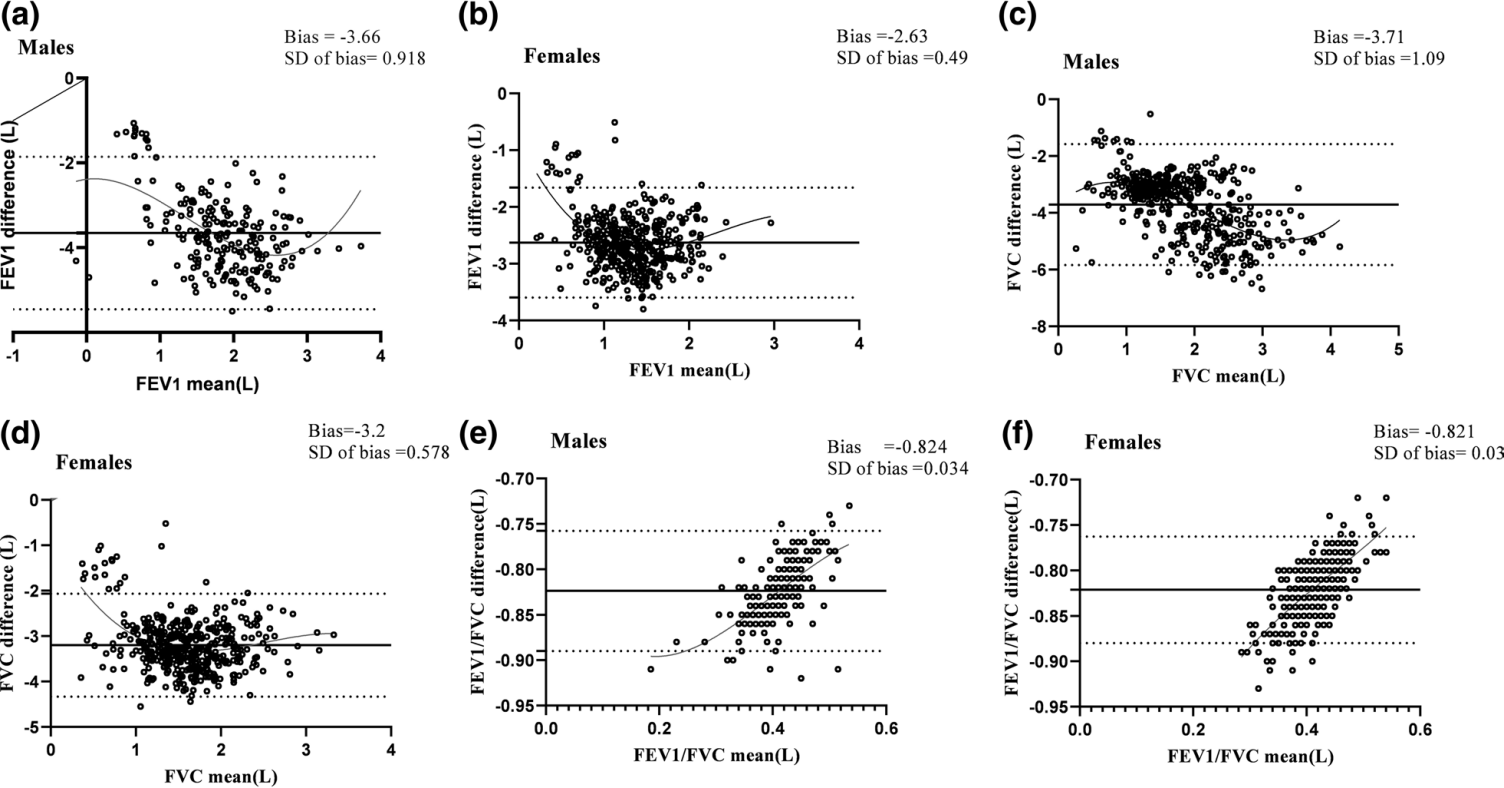
The Bland-Altman plots of FEV1, FVC and FEV_1_/FVC by gender

The average differences (SDs) in FEV_1_ (L), FVC and FEV_1_/FVC (%) predictions were − 3.66(0.918), − 3.7 (1.087), and – 0.824 (0.034) for men, and − 2.63 (0.49), − 3.2(0.57), and − 0.821 (0.003) for women, respectively. The Bland-Altman plots of FEV1 (L), FVC and FEV1/FVC (%) are shown in Fig. 2a–f.

## Discussion

This is the first study for the Iranian population that derived predictive equations and values using Lambda–Mu–Sigma (LMS) [18] by GAMLSS models. This model is preferable to the conventional multiple regression analysis which limits the model to several assumptions including normality of the residuals and constant variance [24]. On the other hand, LMS provides a variation in computing LLN through anthropometric data and prevents under-diagnosis of abnormalities in younger and taller individuals, and over-diagnosis of lung disorders in older and shorter people [25].

In this study, we have generated prediction equations for FEV_1_, FVC, FEV_1_/FVC, and FEF_25−75%_ based on lung function data from 622 healthy Iranian populations. Genetic and environmental variables play a substantial role in the variability of lung function, so it is important to establish reference values appropriate to the ethnic and ecological characteristics of the local population [26, 27].

Our findings showed that GLI-2012 new equations adequately fitted FEV_1_, FVC, FEV_1_/FVC, and FEF_25−75%_ data on the Iranian population for both genders.

In this population study of lung function, we assessed the agreement of lung function predictions between the GLI-2012 Caucasian values and GLI-2012 Iranian measures. The largest average difference was observed in FVC among men and the lowest difference was related to FEV_1_/FVC index in men and women.

In a study conducted on Jordanian people over 18 years old, based on Bland and Altman results, there were significant differences between the new equation and GLI for Caucasians equations too [24, 28].

In our study, age and height was the main predictors of the FEV_1_ (males), FVC (males and females), and age for FEV_1_/FVC (not height) for both sexes.

In different similar studies on various ethnicities, anthropometric predictors have been measured on spirometric indices in both sexes. In a study conducted in India, it was found that age and height were the main predictors of the FEV_1_ and FVC spirometry parameters in both sexes, for FEV_1_/FVC, only age was a significant predictor of outcome [29] but not height. This result was consistent with the findings of our study. Chang’s and colleagues reported, the height and weight, but not age, were important predictors in the final prediction models for FVC and FEV_1_ in Taiwanese children [30].

In our study, the frequency Z-score of FEV_1_/FVC below LLN was less than 5% in all age groups, except for the group of men over 70 years old (12.5%). This finding was consistent with the results of a study conducted in India 29. But this amount was estimated at 10% in Mozambique’s reference population (Southeast Africa) [31], also the LLNs of FEV_1_/FVC were less than 0.70 in men above 56 years of age and women above 60 years of age in Chinese aged 4–80 years [1]. Concerning the high prevalence in men over 70 years of age, this may be due to the low sample size in this age group (16 people). However, the initial interview to enter the study was accompanied strictly, but the possibility of bias could not be prevented absolutely. For example, some elderly men may have had the experience of smoking in the past but have forgotten or for some reason declare that they have not had this experience.

This study has several limitations. First, the sample size of this study is not very large. However, we would claim that the sample size of men and women is large enough to have enough power for validating spirometry reference values (at least 150 subjects for each gender) [32].

### Conclusion

GLI-2012 Iranian equations fitted FEV_1_, FVC, FEV_1_/FVC, and FEF_25−75%_ data of Iranian population for both gender. There were significant differences between measures by GLI for Caucasians and Iranian (new) equations. It is recommended that the values and equations generated from this study should be used by physicians and experts in practice for detecting the disease condition and its severity in Iranian populations.

## Supplementary Information

**Additional file 1: Fig. S1.** Normal Q–Q plots for FEV_1_, FVC, FEV_1_/FVCand FEF_25–75%_ by gender.

**Additional file 2: Table S1.** The association between spirometric indices and anthropometric parameters.

## Data Availability

The datasets generated and/or analyzed during the current study are available from Dr. Leyla Sahebi (first author) on reasonable request.
