# Supplementary material for: Normal spirometry prediction equations for the Iranian population
Source: BMC Pulm Med. 2022 Dec 12;22:472. doi: 10.1186/s12890-022-02273-8 (PMC9746105; doi:10.1186/s12890-022-02273-8)
Supplement: Supplementary file 1 — Additional file 1: Fig. S1. Normal Q–Q plots for FEV1, FVC, FEV1/FVCand FEF25–75% by gender. [file 12890_2022_2273_MOESM1_ESM.docx]

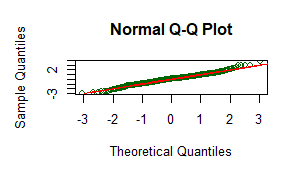

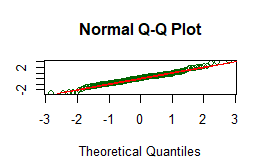


1b: FEV1 in females

1a: FEV1 in males

1d: FVC in females

1c:FVC in males


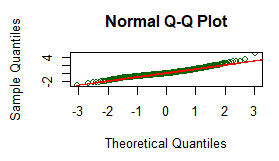

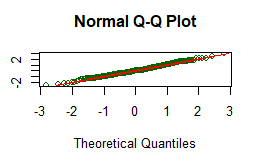


1f: FEV1/FVC in females


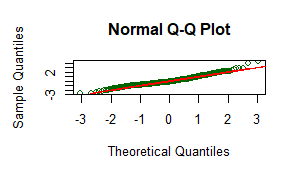

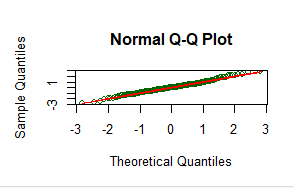


1e: FEV1/FVC in males

1g: FEF25-75% in males

1h: FEF25-75% in females


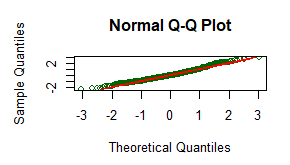

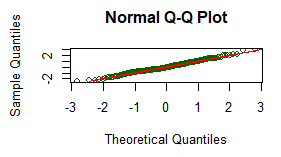


Figure 1a to 1h supplementary: Normal Q-Q plots for FEV_1_, FVC, FEV_1_/FVC and FEF_25-75%_ by gender
