# Supplementary material for: Normal spirometry prediction equations for the Iranian population
Source: BMC Pulm Med. 2022 Dec 12;22:472. doi: 10.1186/s12890-022-02273-8 (PMC9746105; doi:10.1186/s12890-022-02273-8)
Supplement: Supplementary file 2 — Additional file 2: Table S1. The association between spirometric indices and anthropometric parameters. [file 12890_2022_2273_MOESM2_ESM.docx]

**Table S1**: The association between spirometric indices and anthropometric parameters

| Spirometry indices | Anthropometric indices | R squared in males | | R squared in females | |
| --- | --- | --- | --- | --- | --- |
|  |  | LLN | Predicted value | LLN | Predicted value |
| FEV_1_ | Age | 0.84 | 0.78 | 0.87 | 0.42 |
|  | Height | 0.69 | 0.85 | 0.55 | 0.45 |
| FVC | Age | 0.82 | 0.76 | 0.85 | 0.78 |
|  | Height | 0.78 | 0.89 | 0.61 | 0.79 |
| FEV_1_/FVC | Age | 0.87 | 0.59 | 0.79 | 0.82 |
|  | Height | 0.15 | 0.29 | 0.13 | 0.16 |

R squared by nonlinear correlation (cubic correlation)
